# Exploring the effect of UV-C radiation on earthworm and understanding its genomic integrity in the context of H2AX expression

**DOI:** 10.1038/s41598-020-77719-2

**Published:** 2020-12-03

**Authors:** Karthikeyan Subbiahanadar Chelladurai, Jackson Durairaj Selvan Christyraj, Ananthaselvam Azhagesan, Vennila Devi Paulraj, Muralidharan Jothimani, Beryl Vedha Yesudhason, Niranjan Chellathurai Vasantha, Mijithra Ganesan, Kamarajan Rajagopalan, Saravanakumar Venkatachalam, Johnson Benedict, Jemima Kamalapriya John Samuel, Johnson Retnaraj Samuel Selvan Christyraj

**Affiliations:** 1 Regeneration and Stem Cell Biology Lab, Centre for Molecular and Nanomedical Sciences, International Research Centre, Sathyabama Institute of Science and Technology, Chennai, 600119 Tamilnadu India; 2 Present Address: Centre for Nanobiotechnology, Vellore Institute of Technology, Vellore, 632014 Tamilnadu India; 3 Present Address: Department of Bioinformatics, Science Campus, Alagappa University, Karaikudi, 630004 Tamilnadu India; 4 Department of Biotechnology, Anna University of Technology, Tiruchirappalli, 620024 Tamilnadu India

**Keywords:** DNA damage response, Environmental monitoring

## Abstract

Maintaining genomic stability is inevitable for organism survival and it is challenged by mutagenic agents, which include ultraviolet (UV) radiation. Whenever DNA damage occurs, it is sensed by DNA-repairing proteins and thereby performing the DNA-repair mechanism. Specifically, in response to DNA damage, H2AX is a key protein involved in initiating the DNA-repair processes. In this present study, we investigate the effect of UV-C on earthworm, *Perionyx excavatus* and analyzed the DNA-damage response. Briefly, we expose the worms to different doses of UV-C and find that worms are highly sensitive to UV-C. As a primary response, earthworms produce coelomic fluid followed by autotomy. However, tissue inflammation followed by death is observed when we expose worm to increased doses of UV-C. In particular, UV-C promotes damages in skin layers and on the contrary, it mediates the chloragogen and epithelial outgrowth in intestinal tissues. Furthermore, UV-C promotes DNA damages followed by upregulation of H2AX on dose-dependent manner. Our finding confirms DNA damage caused by UV-C is directly proportional to the expression of H2AX. In short, we conclude that H2AX is present in the invertebrate earthworm, which plays an evolutionarily conserved role in DNA damage event as like that in higher animals.

## Introduction

For the survival of organisms, maintenance of genome integrity is crucial and inevitable^1^. In the context of evolution, higher organisms have developed complex mechanisms to identify and repair DNA breaks^2,3^. The DNA damage engages with a series of cellular response events that result in either cell cycle arrest or in some extreme cases it leads to apoptosis^4^. When the DNA damage occurs, its hurried and synchronized action of various signaling pathways, including cell-cycle checkpoint activation^5,6^, histone modification near the site of the break^7^, chromatin remodeling^8^, cohesins modulation^9^, and activation of DNA-repair proteins^6^. Many of the defects in these pathways lead to several disorders in human^5^. The genome integrity is challenged by several factors and one of the main factors is UV radiation^10^. Therefore, studies on UV radiation on biological system is getting more attention.

Ultraviolet (UV) is one of the components of solar radiation, which is classified into UV-A (320–400 nm), UV-B (280–320 nm), and UV-C (200–280 nm)^11^. The UV-C and most of the UV-B do not have the ability to reach Earth because it is stopped by the stratospheric ozone layer. Therefore, only UV-A and slight UV-B reach the Earth^12^. As reported already, the total stratospheric ozone layer depleted by 10% so that the UV-B reaching the Earth’s surface increased by 20%^13^. However, a recent study shows that there is some recovery from ozone depletion; but that is not significant except in Antarctica^14^. Although UV-C has not reached the Earth’s surface from the solar light, many man-made activities like arc-welding torches^15^, mercury lamps, UV sanitizing bulbs^16^ emit UV-C. Similarly, UV-C is highly used for post-harvest improvement^17^ and also used in food industries as disinfection^18^. Even in the COVID-19 pandemic, people are suggesting to use low dose of UV-C (222 nm) to reduce the ambient level of airborne viruses with positive results coming out in in-vitro experiments^19^; but how safe it is in the real-world environment is questionable. UV-A light is poorly absorbed by most biomolecules; however, it can damage DNA indirectly through the photosensitizer molecules. UV-B is directly absorbed by nucleic acids and generates reactive oxygen species, oxidative stress, and inducing DNA damage. UV-C is also strappingly absorbed by nucleic acids of purine as well as pyrimidine bases and generate excited-state species, which leads to DNA damage and results in cell death or mutation^20,21^.

H2A histone family member X is a type of histone protein and referred to as H2AX. The phosphorylated H2AX (γH2AX) foci formation is a potent tool used to study DNA double standard break formation and repair after genomic damage, change in chromosome dynamics, and signalling mechanisms associated with DNA damage response^22,23^. H2AX is highly conserved from *Giardia intestinalis* to *Homo sapiens*^24^. Reports clearly suggest that UV exposure induces H2AX^25^. In addition, solar radiation effects on the living system have been highly discussed^26,27^; but limited studies discussed the effect of UV-C in animals. Concerns with high depletion of the ozone layer and increasing uses of artificial UV-C lead us to showcase the importance of UV-C effect on the animal system.

Earthworms are hermaphrodite, segmented, tubular worm, which come under phylum-Annelida, class-Oligochaeta^28^. These are widely used as a model system for regeneration^29^ and toxicology studies, worldwide^30^. In the present study, we investigated the effect of UV-C on earthworm, *Perionyx excavatus* and its impact on genome integrity. In addition, we explored the presence of H2AX protein in earthworm and confirm its role in DNA repairing event.

## Results

### Effect of UV-C on earthworm behavior

**Figure 1:**
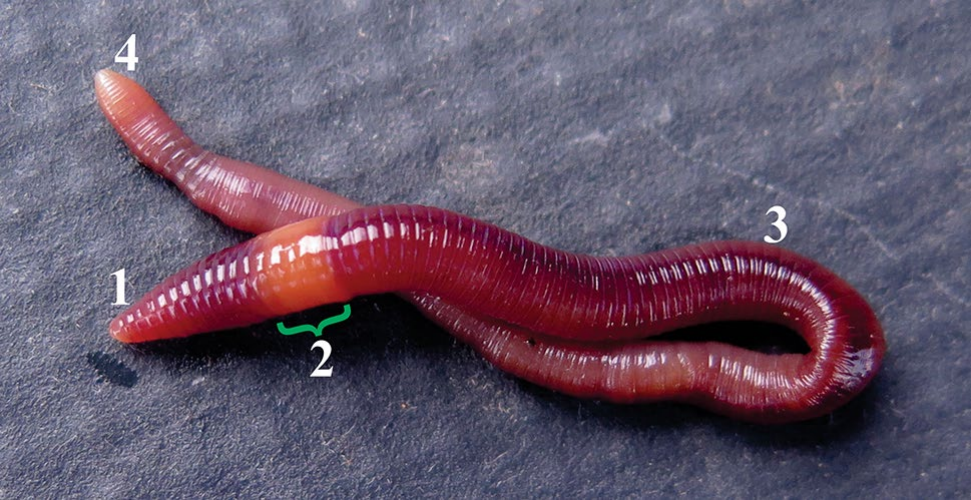
*Morphology of earthworm, P. excavatus. *The anterior region of the worm (1 to 12th segments) is called as head, which comprises of prostomium, brain, heart, seminal vesicles, testis (1), Muscular thick portion that occurs behind the head region is called as clitellum and it is located in between 13–17th segment (2), Following clitellum region, there are no notable organ system present in the post clitellum region except a pair of prostate gland that lies on 18th segment (3). The posterior end of the worm is called tail region (4).

**Figure 2:**
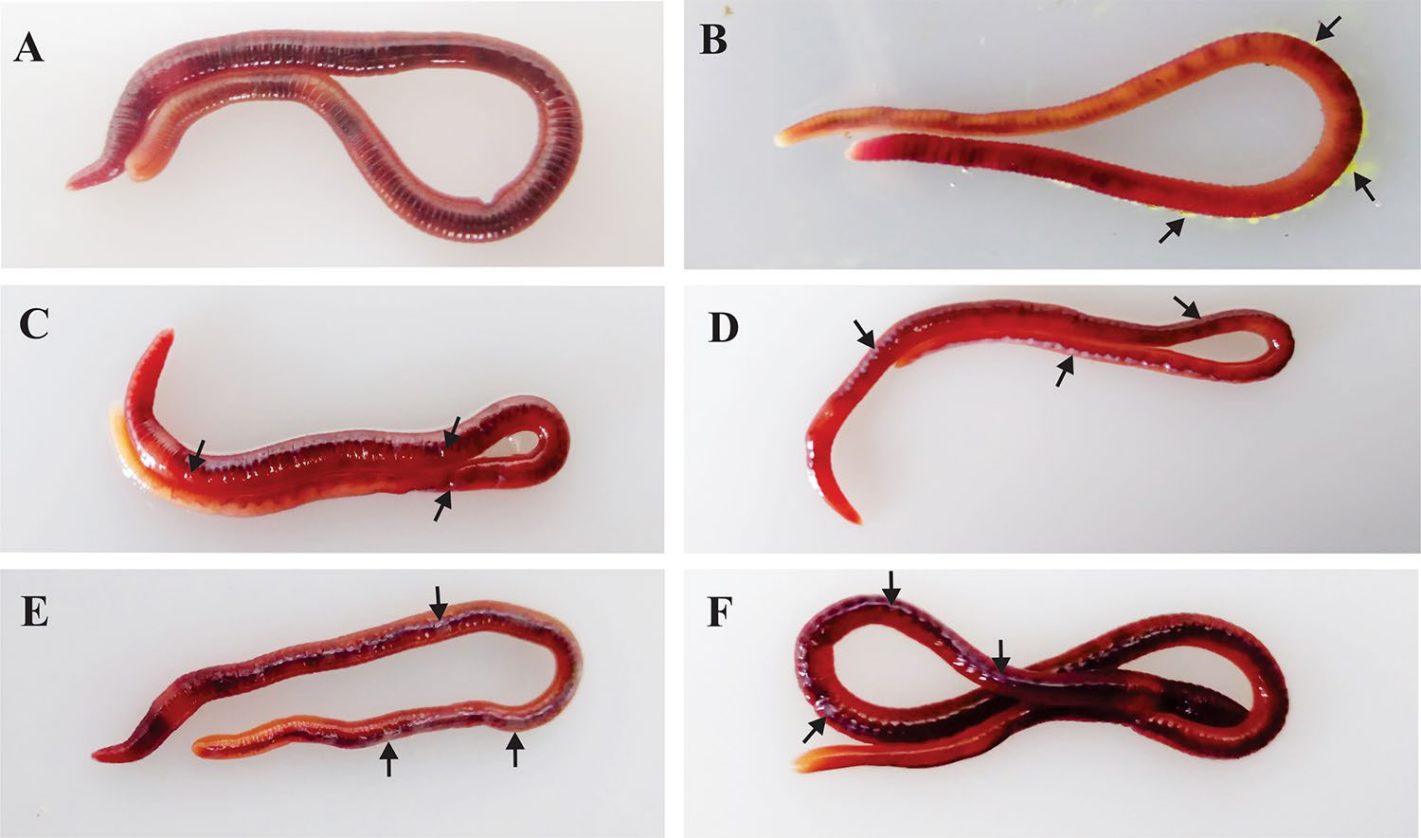
Releasing of earthworm coelomic fluid in different condition. (**A**) Control worms without stress not releasing coelomic fluid on the dorsal surface. (**B**) Releasing of coelomic fluid in 10% ethanol. (**C**) Releasing of coelomic fluid after 5 min of UV-C treatment. (**D**–**F**) White color patch of coelomic fluid visible after 15 h of post UV-C exposure of 2, 3, and 5 min, respectively.

Earthworms are used as an animal model for the current experimental setup as described in materials and methods section. *P. excavatus* like other species of earthworm has a segmented body including head, clitellum, post clitellum and tail region (Fig. 1), and it looks smooth and shiny (Figs. 1,2A). Before proceeding with the experiments, it is essential to know the behavior of the earthworm under daylight and night conditions. In the night condition, the crawling speed of *P. excavatus* is very slow and noted as relaxed crawling. But under the daylight condition at laminar airflow, it looks slightly stressed and the crawling speed is slightly higher when compared to the night condition. Interestingly, if you switch off the normal light and switch on the UV-C light, it shows the vigorous altered behavior with a lift up of one-fifth of their body from the platform and shaking it (Supplementary Video S1). Generally, whenever they gets stressed, they start to release a thick yellow colored coelomic fluid through their dorsal pores (Fig. 2B). Notably, earthworm crawling speed doubles when they are exposed to increasing dose of UV light; but once it is over the optimal tolerance level, that is, UV exposure dose of 5 min hinders their active crawling along with secretion of a flimsy, colorless coelomic fluid around their dorsal surface (Fig. 2C). However, it is visible in white color patches following a 15 h post UV-C treatment, which shows the increasing appearance on the dorsal skin respectively from 2 min (Fig. 2D), 3 min (Fig. 2E), and 5 min (Fig. 2F). But the similar formation of white color patches on the dorsal surface of the worm is not observed in control and 1 min UV-C exposed worms.

### Survival ability of earthworm following UV-C exposure

**Figure 3:**
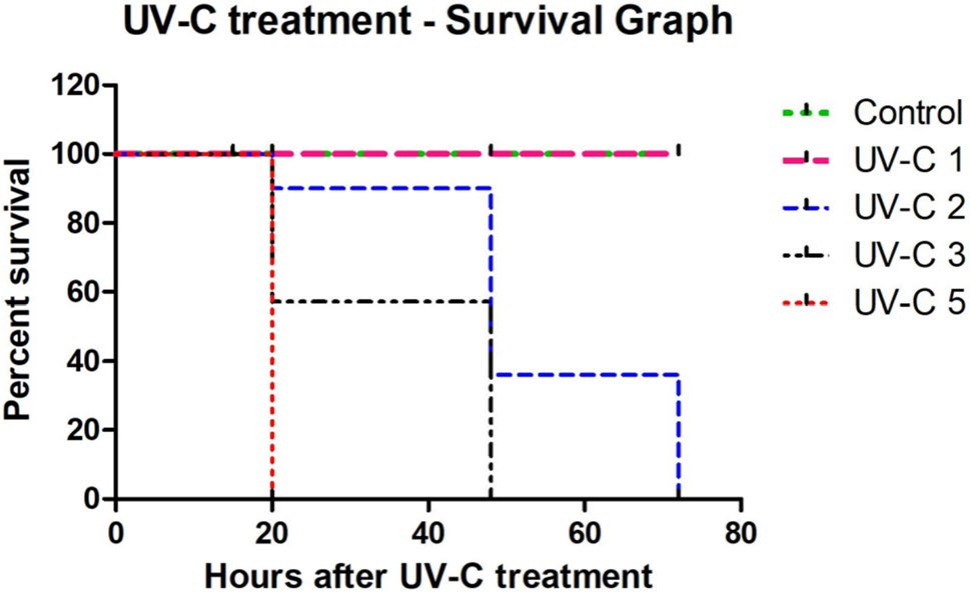
Mortality rate after 20 h of post-UV-C treatment at various doses. No individual worms died in the control group. When the earthworms are exposed to 1 min of UV-C exposure, no mortality is observed. But after exposure to higher doses of (2, 3, and 5 min) exposure, it shows the mortality rate of 20%, 40%, and 100%, respectively.

All the groups of UV treated worms (1, 2, 3, and 5 min) survive till 15 h of post-UV-C treatment. But after 15–20 h of post-UV treatment, we can observe worm mortality and notable skin damage. In 1 min UV-C-treated group, slight skin burns are visible in 1/10 worms, but all other worms are alive and active. In 2 min UV-C-treated group, 2/10 worms die and another 2/10 worms are highly damaged in the outer tissue layer and the remaining 6/10 worms are alive. In 3 min UV-C-treated group, 4/10 worms die, 2/10 worms are highly damaged in the outer skin layer, and the remaining 4/10 worms are alive. But we observe 100% mortality in 5 min UV-C-treated worms after 20 h of post UV exposure (Fig. 3). Similarly, the entire group of worms in 2 min and 3 min UV-treated groups died within 72 and 48 h, respectively. The 1 min UV-C-treated worms show 100% UV tolerance without any mortality similar to control group.

### Phenotypic observation

**Figure 4:**
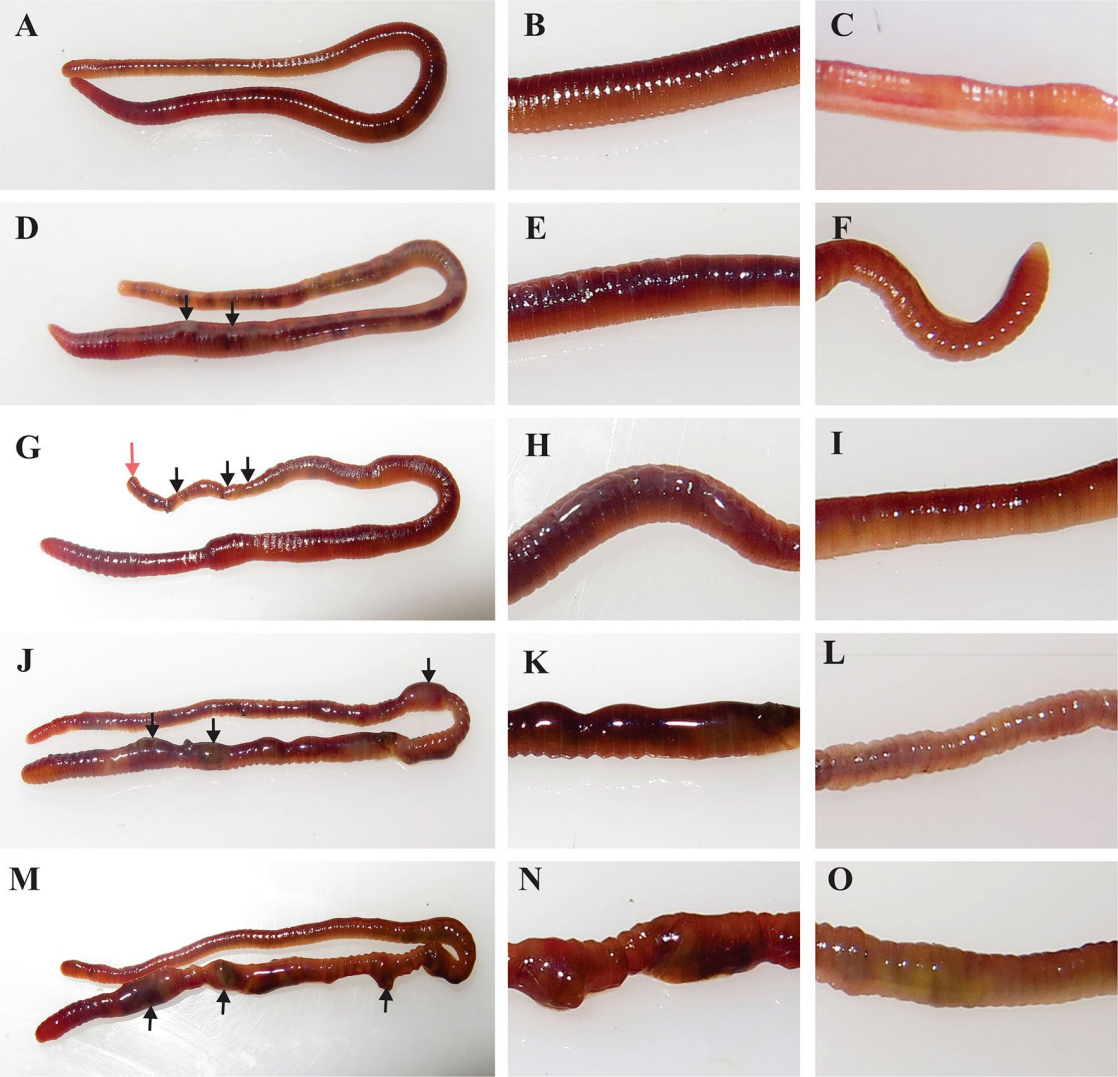
Phenotypic changes in the earthworm, *P. excavatus* after 20 h of post-UV-C exposure. (**A**–**C**), **(D**–**F**), (**G**–**I**), (**J**–**L**) and (**M**–**O**) respectively from control, 1, 2, 3, and 5 min UV-C-treated worms. Control worms without exposure to UV-C (**A**–**C**). Slight burn occur in UV-C, 1 min treated worms (**D**, **E**); autotomy (red arrow), and cleavage furrow (black arrow) visible in UV-C, 2 min treated worms (**G**); tissue inflammation visible in UV-C, 3 min treated worms (**J**, **K**); High inflammatory, tissue segments (dead worm) appear in UV-C, 5 min treated worms (**M**, **N**). No tissue damage observed in control post clitellum region (**B**). The damage is clearly visible in the post clitellum region and it increases from 1, 2, 3 and 5 min UV-C treated worms respectivily **E**, **H**, **K** and **N**. No color change in control group tail (**C**). The yellow color appears and gradually increases in the tail region of the 1, 2, 3, 5 min UV-C treated worms respectivily  from **F**, **I**, **L** and **O**.

**Figure 5:**
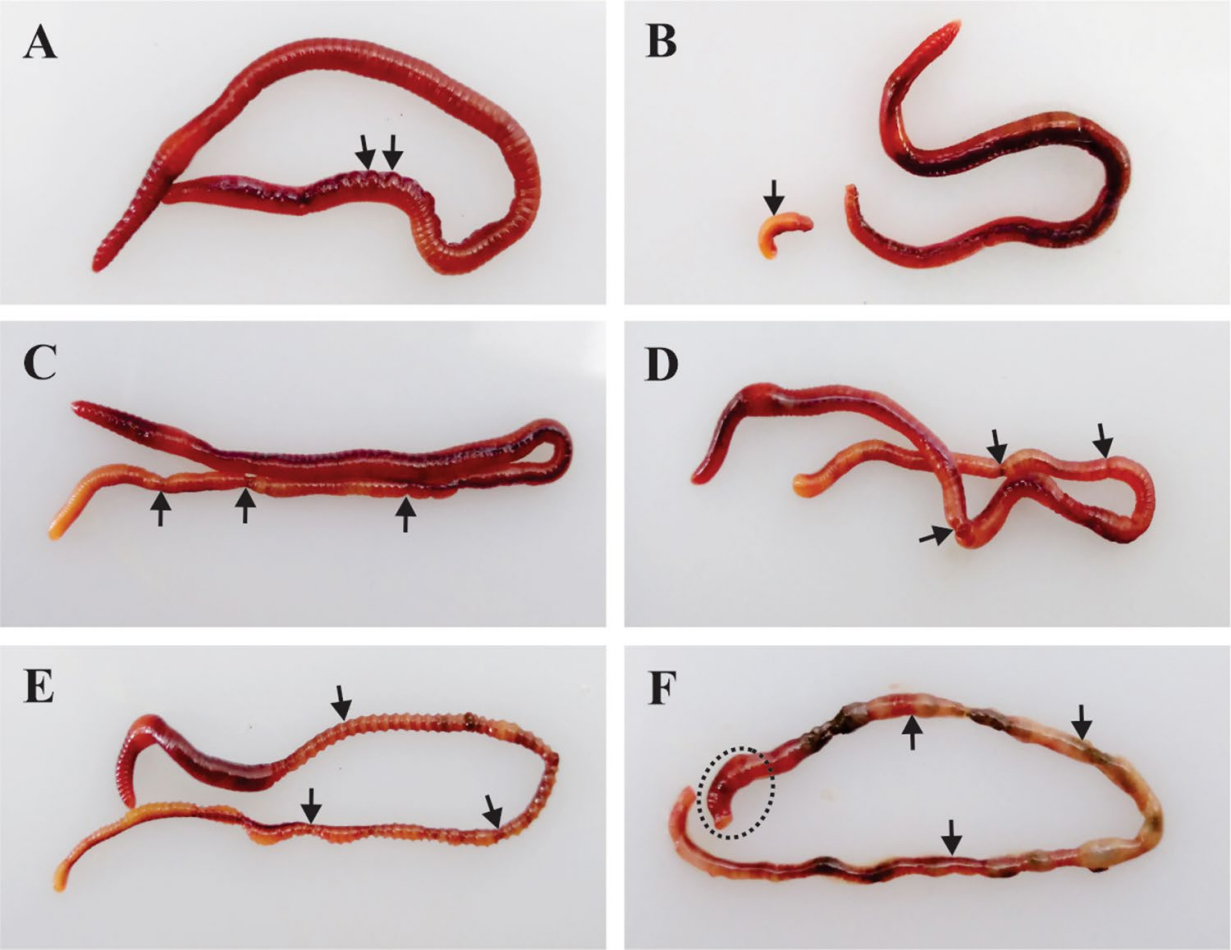
Phenotypic changes observed in earthworm, *P. excavatus.* After 20 h of post-UV-C exposure, tissue bulges are observed in some segments (**A**). Small pieces of tail segments are separated from the worm by autotomy (**B**). Cleavage furrow appears in the post clitellum region (**C**). Cleavage furrow almost proceeds with formation of detached segments (**D**). In the post clitellum region, the worm segment is fully dehydrated (**E**). Dead worms fully degenerated in the posterior region, but not in the anterior region from mouth to clitellum segments; but prostomium gets damaged heavily (**F**).

After 20 h of post UV treatment, except in the control worm (Fig. 4A–C), phenotypic changes are observed in all other groups of worms. In 1 min UV-treated group, 1/10 worms show slight skin burn (Fig. 4D,E). But in all other groups (2, 3, and 5 min UV-exposed worms), distinct phenotypic changes are observed as shown in Fig. 4G–O. In 2 min UV-treated worms, the following notable characteristic changes are observed: (1) Skin burns observed in all the worms (Fig. 4G,H); (2) Segment swellings observed in 1/10 worms (Fig. 5A); (3) Cleavage furrow formation in 4/10 worms (Figs. 4G,5C); and (4) Damaged tail detaches by autotomy 1/10 worms (Figs. 4G,5B). In 3 min UV-C-treated worms, tissue inflammation is observed in post-clitellum segments with distinguished tissue color change from brownish to slight yellow in 6/10 worms (Fig. 4J,K). In 2/10 worms, the cleavage furrow formed in the post-clitellum segment proceed further to form detached segments (Fig. 5D). In 5 min UV-treated group, severe tissue inflammation is observed in the post-clitellum segments with a distinct knot-like structure formation in 8/10 worms (Fig. 4M,N). In the knot formation site, the brownish skin color turns to blackish pale yellow color. Another interesting observation noticed is in the tail region of the UV-C-treated worm where the tissue color gradually changes from pale yellow to greenish yellow, which is more prominently visible in 5 min-treated group (Fig. 4C,F,I,L,O). The crawling speed of the worms slowdown in the 2 and 3 min UV-C-treated group with high incidence of slow crawling observed in highly damaged. Similarly, in 3 and 5 min UV-C-treated groups, 3/10 and 2/10 worms observe high weight loss along with dehydrated tissue nature (Fig. 5E). The knot formation and degeneration occur in 5 min UV-C-treated group at posterior segments, but no such changes are visible in the anterior region from prostomium to clitellum region (Fig. 5F).

### Pathological effects of UV-C

**Figure 6:**
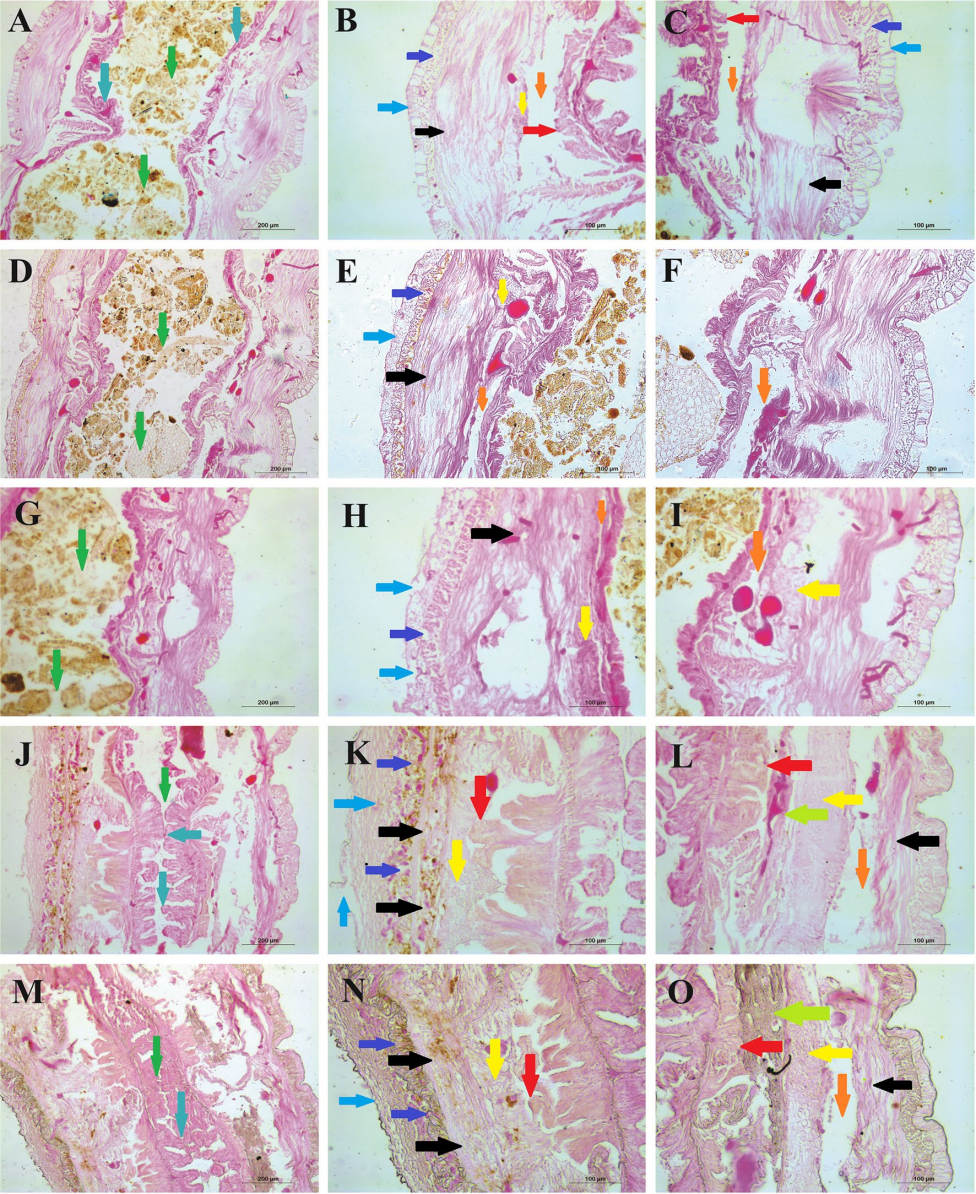
Longitudinal tissue structure of *P. excavatus* after 20 h of different doses of UV exposure (post clitellum section—figure panel (**A**), (**D**), (**G**), (**J**), and (**M**)—200 µm; dorsal side—figure panel (**B**), (**E**), (**H**), (**K**), and (**N**)—100 µm; ventral side—figure panel (**C**), (**F**), (**I**), (**L**), and (**O**)—100 µm). (Arrows green—intestinal cavity, blue—intestinal epithelium, sky blue—skin epithelium, violet—circular muscle, black—longitudinal tissue, yellow—coelomocyte, orange—coelomic fluid cavity, red—chloragogen tissue, fluorescent green—unknown structure). Longitudinal sections (LS) of *P. excavatus* without UV-C exposure (**A**–**C**) clearly show all the tissue structures without any damage. In 1 min UV-C-treated worms (**D**–**F**), (**D)**—the visible space in the intestinal cavity, (**E**)—flip structure collapsed in epithelial layer others are similar to (**B**), (**F**)—no notable changes in ventral side similar with (**C**). The 2 min-treated worms show (**G**–**I**) (**G**)—similar to control, (**H**)—high damage in epithelial tissue and slight damage in circular muscle tissue, slight increase in size in the longitudinal tissue layer, no substantial space of coelomic fluid free flow space, (**I**)—reduction of coelomic fluid cavity and slightly increased accumulation of coelomocytes. UV-C for 3 min (**J**–**L**), (**J**)—highly reduced intestinal cavity space, overgrowth of intestinal epithelial tissues, (**K**)—fully collapsed and increased structure of epithelial, slight structural changes and damages of circular muscle, change in tissue pattern and reduction in size of longitudinal tissue layer, high production or accumulation of coelomocyte, substantial growth of chloragogen tissue layer, (**L**)—in ventral side, chloragogen tissue overgrowth, unknown structure (fluorescent green), accumulation of coelomocyte, coelomic fluid cavity space reduced the size of longitudinal tissue layer. Similarly, in 5 min UV-C-treated group (**M**–**O**), (**M**)—intestinal cavity fully blacked, intestinal epithelial fused from both side, (**N**)—dorsal side is similar to (**K**), (**O**)—ventral side is similar with (**L**).

Histological sectioning of control worm tissue architecture show no tissue damage in intestinal cavity, intestinal epithelial layer (Fig. 6A) and in three skin layers, namely epidermal layer, circular muscle, and longitudinal layer. Additionally, the coelomic cavity, which occurs in between intestinal tissue and the skin tissue layers shows free-flow space and slight accumulation of coelomocytes (Fig. 6B,C). In 1 min UV-C-treated groups, no substantial damage or change is observed (Fig. 6D–F) when compared to control, but in the dorsal side of the skin, the epithelial flip structure is disturbed notably (Fig. 6E). However, in 2 min UV-C-treated group, the coelomic fluid cavity is highly reduced (Fig. 6G–I); the epidermis is highly damaged with collapsed flip structure; circular muscle layer shows slight damage and increased tissue pattern of longitudinal tissue layer on the dorsal side (Fig. 6H). Moreover, notable coelomocyte accumulation is observed in both dorsal and ventral side (Fig. 6H,I). Interestingly, in 3 min UV-C-treated group, the intestine gets shrunk, no space is observed in between the adjacent intestinal epithelium and thereby completely blocking the passage of food (Fig. 6J). Additionally, the outgrowth of chloragogen tissue layer at the dorsal side pushes the coelomocyte towards the damaged skin layer and thereby seals the entire tissue layer and blocks the free flow of coelomic fluid (Fig. 6J,K). Overall, the dorsal side of the worm is highly exposed to UV-C and receives more damage with an enlarged epithelial layer together with the collapsed flip structure. UV-C-induced damages are commonly observed in the circular muscle layer with collapsed structure. In high UV-C dose, reduced tissue pattern of longitudinal skin layer is also observed. The skin layer together with chloragogen tissue and coelomic fluid make it a condensed stagnated structure (Fig. 6K). Similarly, at the ventral side, stagnant or condensed coelomic fluid towards the chloragogen tissue layer is visible and notably the coelomic fluid free flow space in between ventral skin tissue and the longitudinal layer is highly reduced (Fig. 6L). Similarly, in 5 min UV-treated group, the inner side of intestinal epithelial tissues fuses at many sites preventing the free flow of food (soil). The outer face of intestinal tissue outgrowth merges with the overlaying longitudinal cell layers along with the coelomocytes (Fig. 6M). The dorsal and ventral sides of the 5 min-treated group are similar with the 3 min-treated group (Fig. 6N,M). However, there is an unknown visible structure observed (Fluorescent green arrow) in-between the intestinal tissue and the accumulated coelomocytes of the ventral side of the worm (Fig. 6L,O).

### UV-C effect on earthworm genome integrity

**Figure 7:**
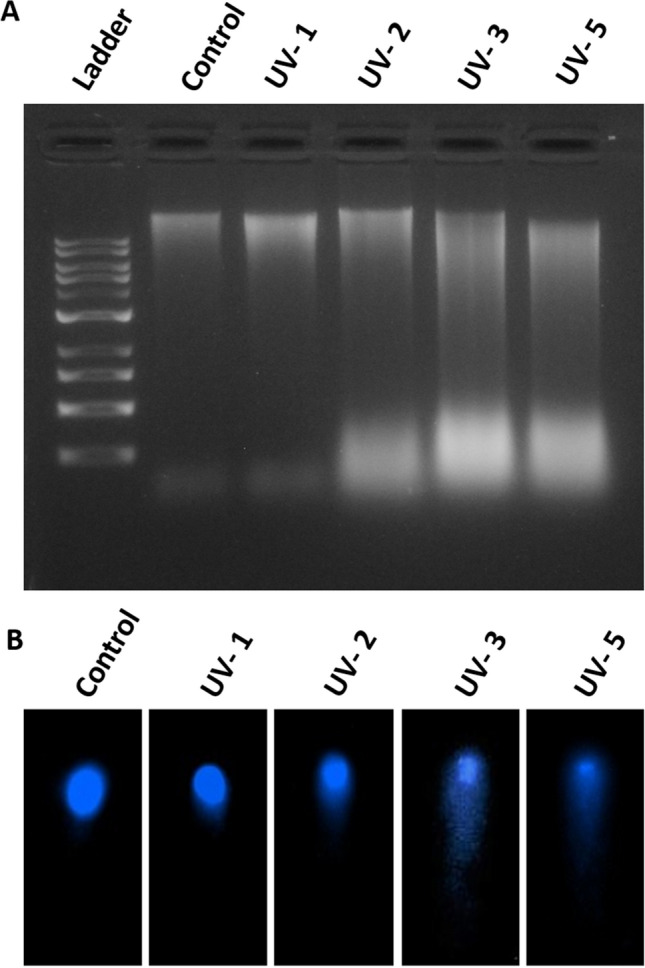
DNA fragmentation assay and Comet Assay. (**A**) DNA fragmentation assay—ladder (1 kb), Control group DNA with a single clear band and from 1, 2, 3, and 5 min UV-C-exposed earthworms, DNA are fragmented and come with dragged structure. The DNA fragmentation dragged is high in UV-C 3 and 5 min-treated g**r**oups. (**B**) Comet assay—in control group, the DNA appears in a proper round structure whereas in 1–5 min UV-C-treated group, it shows increased tail movement.

The genome integrity of the earthworm is analyzed by DNA fragmentation assay. DNA is isolated from UV-C-treated and the control group of earthworms and resolved in agarose gel electrophoresis. In the control group, genomic DNA is clearly visible as a single band, but in the UV-C-treated groups, DNA fragments are visible as a sheared band because of the fragmented DNA. The fragmentation gradually increases from UV-C 1 min- to UV-C 5 min-treated group. The DNA fragmentation is high in the UV-C 3 min- and 5 min-treated group (Fig. 7A). The data confirms that the earthworm DNA damage is increased by increasing the UV-C dose. Further to confirm the UV-C-induced DNA damage, alkaline comet assay is performed using the DNA that are isolated from the UV-C treated earthworm tissue, which shows DNA damage increases in a dose-dependent manner which are revealed through the increasing size of the comet length. In control, the DNA is visible as circular in structure, but in 1 min-treated group it shows a slight halo structure around the comet head. Moreover, very few comet tails are visible in the same group. However, comet tail gradually increases from 2 min, 3 min, and 5 min UV-C-treated group (Fig. 7B) in parallel to the decrease in comet head. From the results it is evident that DNA single strand break and increased length of tail, which referred to the high DNA damage. These long comet tails are observed more in number in UV-C 3 and 5 min-treated animals.

### Effect of UV-C on H2AX and β-actin

**Figure 8:**
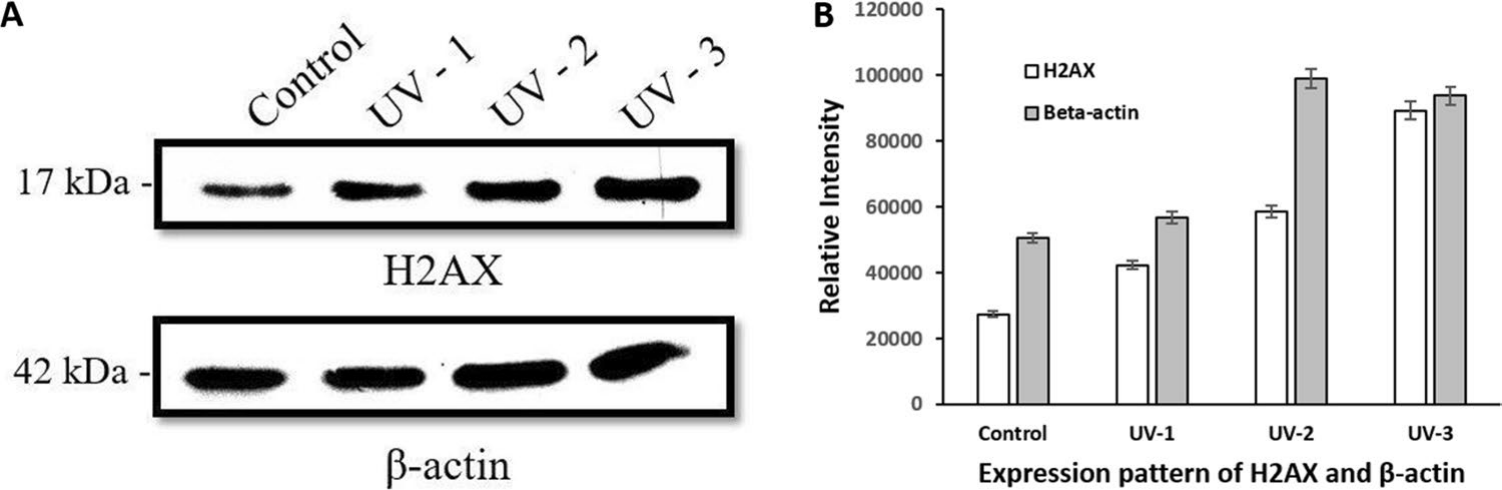
H2AX and β-actin expression in the earthworm, *P. excavatus* after 20 h of post UV-C exposure—(**A**) Western blot analysis, H2AX expression is gradually increased in 1 and 2 min UV-C exposed earthworms; in 3 min UV-C-treated group double the expression of H2AX was observed when compared with the control group. Similarly, β-actin, the reference protein also shows upregulated expression as the dose of UV-C increases. (**B**) Quantification of H2AX and β-actin expression are done based on the band intensity and represented using bar diagram. To analyse the statistical significance, the experiments were repeated in triplicate and their value is represented as mean ± SD. *p*-value < 0.05 was considered as a statistically significant data.

To study the expression of H2AX and β-actin following UV-C treatment, Western blot analysis is performed. Briefly, protein lysate is prepared from the control and UV-C-treated earthworms and subject to Western blotting against H2AX and β-actin antibodies. The data shown in Fig. 8A proves that upon exposure to increasing dose of UV-C, *P. excavatus* show gradual upregulation of H2AX expression from UV-C 1 min-treated to UV-C 3 min-treated groups. H2AX in the 1, 2- and 3-min UV-C-treated groups is overexpressed to a fold of 1.54, 2.14 and 3.26 times higher respectively than the control group (Fig. 8B). Similarly, β-actin, normally used as a loading control is also elevated in UV-C-treated groups. The expression level of β-actin is increased to 1.12, 1.96, 1.85 folds in UV- 1, 2, 3 min treated group respectively than in the control worm (Fig. 8B). Protein sample is not obtained from the 5 min-treated group because no more worms are alive in 5 min UV-C treated group following 20 h.

## Discussion

### Significance of coelomic fluid in earthworm survival

In this present study, the earthworm *P. excavatus* is used as an animal model to study the impact of UV-C on earthworm morphology and genomic integrity. Generally, if earthworms are under stress condition, they release coelomic fluid, which usually possesses several immune defence-related biological functions^31^. The earthworm, *P. excavatus* coelomic fluid is yellow in color due to the presence of autofluorescence riboflavin in the type of coelomocyte called eleocytes^32^ and the viscosity of the fluid is very high (Fig. 2B), which may facilitate more protection comparatively. But on increasing the UV-C exposure time (5 min), the nature of the exorcizing coelomic fluid changes results in secreting flimsy, colorless coelomic fluid (Fig. 2C), which appears as white color patches on the dorsal surface following 15 h of post UV-C treatment (Fig. 2D–F) is noted. The secretion of coelomic fluid is protective to certain extent but increasing the UV-C dose is not able to protect their skin from burn and subsequent death occurs. In human and some animals following UV irradiation, high amount of mediators is released through the skin, which include eicosanoids (PGE2, PGD2, PGF2a, LTB-4, 12-HETE), cytokines ( IL-1, IL-6, IL-8, TNF-alpha), growth factors ( TGF-beta, VEGF, NGF), vasoactive amines, and neuropeptides (histamine, bradykinin, CGRP)^33^. Some report suggesting that some chemicals and enzymes protect the organism from UV radiations; such as mycosporine-like amino acid and flavonol quercetin or photolyase^34–36^.

Following exposure to UV-C, the ventral side of earthworm becomes slight yellowish when compared to control worms (Fig. 4C,F,I,L,O). It can be due to the increase in production of coelomic fluid and their stagnation at the site of damages. In earthworm, respiration is depending on the network of small blood vessels present in the body wall epidermis. The oxygen is taken from the moist surface of the body wall, which keeps moisture by mucous gland of the epidermis^28^. When exposed to increased dose of UV-C, the epidermis and mucous gland get damaged, which makes it difficult to respire and leads to suffocation. Likewise, coelomic fluid circulation mainly depends on muscle contraction and the damaged epithelium causes abnormal muscle contraction that also leads to suffocation^37^. While exposing to UV-C, we observe the vigorous behavior of earthworm and the worm tries to avoid the UV exposure, possibly due to the effect on sensory neurons. Similarly, zebrafish larvae also have negative phototaxis when exposed to UV light^38^. In the earthworm body, there are nerve fibers and nerve plexus (motor neuron and sensory neuron), which arise from the segmental neuron. Specifically, nerve plexus occurs in the epidermis and it is well observed that the sensory cell of the epidermis transmits impulses directly to muscles as well as to the central nervous system of earthworm^39^. Hence, UV-C radiation effects on motor neuron leads to muscle constrain. In the meantime, muscular coordination between the circular and longitudinal layer is very essential for proper muscular contraction^37^ and in UV-C-exposed worms, the circular and longitudinal layers are damaged, which directly affects the crawling of earthworm. Collectively, upon increasing UV-C dose, the amount of coelomic fluid released is gradually increased and at the same time tissue respiration is highly affected.

### Tissue response and assessment of autotomy

Many studies report the effects of UV radiation on several organisms^40–42^. In particular on earthworm, Chuang et al. in 2006 reported that the earthworm, *Amynthas gracilis* tolerate UV-A, dies when exposed to UV-B, and shows lower tolerance than *Metaphire posthuma* species when exposed to UV-B. But *Pontoscolex corethrurus* species show the highest tolerance and survival ability even at a high dose of UV-B^37^. In our present study, *P. excavatus* survive up to 15 h of post-UV-C treatment in all groups, but after 20 h of post UV treatment, damages are visible, which is directly proportional to the exposure dose of UV-C. The formation of white patches on the dorsal surface of the skin following UV-C exposure are commonly observed in all groups, except in 1 min UV-C-treated earthworms. Similarly, the zebrafish produces a systemic anti-inflammatory and strong proinflammatory responses upon UV radiation^43^. However, the link between UV-B and immunity is reported in mice that UV-B suppresses the immune system by inhibiting memory and effector T-cells^44^. It also prevents atherosclerosis by regulating immuno-inflammatory reactions^45^. In earthworm, immune cells are loaded in the coelomic fluid and its increasing secretion following UV-C exposure definitely triggers inflammatory responses associated with damage of cells. However, the mechanism of earthworm immunity needs to be thoroughly studied.

Several reports have shown that UV induces production of reactive oxygen species, photo-oxidation, and increase in the oxidative damage to biomolecules^46–48^. Earthworm, *Lumbricus terrestris* secretes a photoreactive sterol and produces singlet following UV-A exposure^49^. Similarly, UV-A induces apoptosis in the central nervous system in a p53-dependent manner^50^. In our study, few worms in 3 min and most of the worms in 5 min UV-treated groups showed knot-like structure formation (Fig. 4J,K,M,N) and it is referred to as inflammation. However, similar kinds of inflammation are not visible in the regions where most of the organs are present, which are located within 3rd to 18th segments which includes clitellum. The organs present in these segments may prevent them from visible UV damage**s** than where no organ is present. There are multiple pathways for regulated cell death^51^. Still, UV-C induces death in earthworms and may perhaps lead by apoptosis and/or necrosis pathways. In Ly-5 murine lymphoma cells, UV-C induces apoptosis rather than necrosis^52^. Several organisms like amphibians, reptiles, fishes, and arthropods have a special kind of defence quality to escape from predators by a special process named autotomy^53^.

Autotomy is also reported in earthworm, *Eudrilus eugeniae* in which it helps to eliminate toxic material from their body. Earthworm accumulates the toxic material at the most end segments of the tail region and detaches them to survive the remaining parts of the body^30^. In our study, the forming of cleavage furrow (Fig. 5C) proceeds further with a detachment of damaged segments (Figs. 4G,5D) and it happens mostly in the post clitellum region (Figs. 4G, 5C,D). Since the exposure of UV-C radiation on earthworm exposes all over the body and causes lethal effect, it is not possible to remove the genotoxic effects through autotomy process. In order to survive, worms can only stretch to detach the segments at the end of the tail region (Figs. 4G,5B); but even after a successful detachment, either part of the worm fails to stay alive, suggesting that earthworm can survive using autotomy against lower toxic chemicals not when completely exposed to genotoxic UV-C. However, its competence of autotomy on genotoxic needs to be evaluated by further pointed UV exposure studies**.**

Notably, we observe that whenever the dose of UV-C increases, the damage is deep and highly visible in their skin tissues. Moreover, we observed outgrowth of intestinal tissue structures, which narrow down the intestine and coelomic cavity free flow spaces in the dorsal side (highly exposed tissue) and accumulate the coelomocytes towards damaged dorsal skin tissue (Fig. 6K,N). Similarly, in ventral side it was towards the intestinal tissue not towards the ventral skin. However, this accumulation of coelomocytes make way for free flow towards longitudinal layer tissue of the ventral side (Fig. 6L,O). In overall, overgrowth of chloragogen establishes increased production of coelomic fluid, these overproduction make more coelomocytes availability towards damaged tissue. Upon UV exposure, overproduction of coelomocytes is reported in sea urchin species^54^ and also in response to wound, the accumulation of coelomocytes occurs near wound sites in earthworm species^55^, which supports our findings. These efforts show that worms attempt to tackle the tissue damage made by the UV-C. In contrast, interestingly this accumulated pressure in the coelomic cavity and intestinal epithelium overgrowth influence to shut the intestinal food path (Fig. 6J,M). In *Metaphire posthuma*, following 36 h of post UV-B exposure, the epidermis is pleated, and some epidermal cells show necrosis. But after 48 h of post exposure, the epidermis is damaged and some circular muscles are deformed^37^. Notably, these effects are dependent on the dose and species tolerance, but in *P. excavatus* earthworm, it initiates deadly effect within a day. Based on our data, the effect of UV-C on earthworm shows obvious impacts and the phenotypical, pathological, and lethal assessment confirms that UV-C is more harmful radiation than any other subdivided UV spectrum.

### The linkage between genome integrity and gene regulation

One of the main effects of radiation exposure is the formation of single strand (ssDNA) and DNA double-strand breaks (DSB) together referred to as DNA damage^56^. High DSB levels can lead to cell death and low levels of DSB result in genomic rearrangements and may lead to cancer. In this present study, DNA fragmentation assay shows a clear picture of DNA damage when earthworms are exposed to certain dose of UV-C (Fig. 7A). Many studies reveal that UV irradiation induces apoptosis^57,58^ and few studies refer comet assay for indication of apoptosis^59^ and some referred alkaline comet assay for single strand DNA break. We performed alkaline comet assay and it further confirms that UV-C induces significant damage to the earthworm DNA in a dose-dependent manner (Fig. 7B). Tissue size of about 10–12 segments is taken for the sample preparation, which include all the tissue layers from both dorsal and ventral side. Therefore, we observed different size of the comet tail structure. But the high number of comet tails are observed in 2 min- and 3 min-treated animals, whereas the long comet tails are observed in 3 min and 5 min-treated groups. However, 1 min-treated group also shows very few small comet tail DNA, which indicates that even in 1 min of UV exposure, it can damage the earthworm DNA and it could be in the outer layer tissue but it does not lead to death of the animal. The number of comet tail DNA in 5 min-treated group is lower than the 3 min-treated group. It might be due to DNA degradation and the DNA degradation during apoptosis is well discussed^60^. An author discusses the importance of DNA fragmentation in apoptosis and also suggests that during DNA fragmentation, DNA DSB can promote efficient apoptosis^61^. However, DNA degradation during UV-induced apoptosis in earthworms needs a detailed study. Through the tissue inflammation (Fig. 4J,K,M,N) and DNA damage (Fig. 7A,B), we infer that the UV-C-induced death in earthworm may be caused by both necrosis and apoptosis pathways.

During apoptosis, the initial DNA fragmentation encourages H2AX phosphorylation^62^. Many reports support the importance of the histone variant H2AX in various other biological processes including cell division, embryonic development, neural stem cell development, and ageing^63^. Mainly H2AX phosphorylation is considered in response to the DNA DSB and ssDNA formation. However, the UV-C that we used in this experiment does not produce DSB directly, but produces cyclobutane pyrimidine dimers (CPDs) and 6–4 photoproducts (6-4PP) majorly^64^. Several reports suggest that H2AX phosphorylation depends on nucleotide excision repair (NER)^65,66^. In addition, UV radiation also generates DSB in an NER-dependent manner^56^ and unrepaired SSB develops into DNA DSB during mitosis^67^ The H2AX phosphorylation in response to DSB formation mediated by the major physiological factor ATM kinase^68^ but for ssDNA formation ATR (the ATM- and Rad3-related protein kinase) phosphorylates the H2AX^69^. But, phosphorylation of H2AX is mediated by all phosphoinositide 3-kinase-related protein kinases (PIKKs), ATM, ATR, and DNA-dependent protein kinase (DNA-PK) in the case of DNA damage caused by radiation^70^ suggesting that radiation causes ssDNA formation and DNA DSB. Although H2AX is largely considered to be a DNA damage marker, UV irradiation induces phosphorylation of H2AX^71^. In human cell lines, γH2AX formation is gradually increased up to 4 h when irradiated with 10–40 J/m^2^ of UV-C^72^. Our result confirms that upregulation of H2AX is directly proportional to the increased level of DNA damages. The combined evidence suggests that DNA fragmentation or damage induced through UV-C radiation causes ssDNA formation and DSB, which leads to apoptosis and phosphorylation of H2AX as the signal for initiation of counteraction. However, increased dose of UV-C associated with increased DNA damage and apoptosis leads to mortality in earthworm.

Several attempts have been made in the past to identify stable and convenient endogenous control genes in human studies. Reports suggest that UV-B irradiation in human skin fibroblasts, β-actin and TUBB1 are used as the reference genes^73^. Hence, we use β-actin as a reference gene for our experiments. Interestingly, we note β-actin gene expression is upregulated and it is visible in 2 min- and 3 min-treated groups (Fig. 8) when compared to control. Many of the reports show that the commonly used reference genes are not stable, the low-dose X-ray irradiation downregulates β-actin^74^; unregulated CDKN1A^75^;18S and B2M genes are unstable under different radiation in human cells^76^. In addition, recent studies show that one of the prominent reference gene, GAPDH expression significantly vary in the earthworm, *Eisenia fetida* under the exposure of UV filter 4-Hydroxybenzophenone^77^. Therefore, housekeeping genes stability varies depending on cellular pathway response to the toxin. From our results, the upregulated expression of β-actin may be due to the need of the cells to maintain the cellular integrity following UV-C exposure.

## Conclusion

Based on behavioral, phenotypical, pathological, lethal effect, DNA damage, and the expression of H2AX analyzed in our study, we conclude that the earthworm, *P. excavatus* is highly sensitive to UV-C. Notably, frequency of those changes increases in the earthworm with an enhanced dose of UV-C. Further, UV-C affects the earthworm system and generate the ssDNA and DNA double strand breaks, thereby disturbing the genome integrity. Our study suggests that UV-C-induced cell death in earthworm is lead by apoptosis and necrosis pathway. Moreover, this study confirms the presence of H2AX protein in earthworm, which shows DNA damage response similar to higher animals like vertebrates. Since earthworms are widely used as an animal model system for regeneration and toxicology studies, our data steps the use of earthworm animal model in radiation research. In addition, we suggest that H2AX can be adopted as a marker for genotoxicity studies. On the other hand, we are focusing to study the influences of UV-C on earthworm regeneration.

## Materials and methods

### Earthworm culture

Earthworm, *P. excavatus* are purchased from ‘Karthik Vermi-compost Industry, Vadipatti, Madurai, India’. Worms are cultured in a plastic container filled with fine soil, cow dung, and leaf litters. Jaggery filtrates are regularly sprayed to the soil for effective culturing of earthworms. The containers are maintained with moisture at ambient temperature around 20–25 °C in a good ventilated room. The worm bed is regularly changed once every month by removing the vermicompost and compensates with soil and leaf litters.

### Ethical statement

The experiments are carried out using lower invertebrate, earthworm therefore ethical statement is not needed. Necessary care is taken in experimental procedure that are intended to avoid unnecessary pain and suffering to the experimental animals.

### UV-C treatment

The mature worms weighing around 300 mg are selected for the present experimental studies. The worms are divided into five experimental groups and each group has ten worms in total. All the group worms are taken in a separate (20 × 18 × 15 cm) plastic tray. To maintain the wet surface, 10 ml of water is filled into the tray so that the tray is wet for few minutes. Following that, the worms are exposed to UV-C for different time periods 1, 2, 3, and 5 min. The UV source is laminar airflow and wavelength of that UV-C light is 253.7 nm and the exposed area is 120 × 60 × 55 cm. Consequently, exposure dose is calculated as 1 min = 1.423 × 10^–17^ J/m^2^, 2 min = 2.85 × 10^–17^ J/m^2^, 3 min = 4.27 × 10^–17^ J/m^2^, 5 min = 7.13 × 10^–17^ J/m^2^ (detailed calculation available in supplementary data 1). But the control groups are not exposed to the UV-C. Following one-time UV-C exposure, all the worms are maintained in a regular bed. After 15th and 20th hours of post UV treatment, the visible physiological changes are analyzed. All the samples are collected from the post clitellum region of earthworm for following experiments.

### Histology

Histology is performed to study the impact of UV-C-treated earthworms by analyzing the cellular structures, morphological changes, and tissue damage. Briefly, worm tissues are fixed with 10% formalin for 24 h. Then gradient (60–100%) isopropyl alcohol is used to dehydrate followed by clearing it with xylene. Following the clearing step, the tissue samples are embedded with paraffin wax. The embedded tissues are sectioned into 6 μm thick sections using a rotary microtome (Leica-RM2125 RTS). Further, the sections are rehydrated and stained with Hematoxylin and Eosin mounted with DPX mounting agent. The results are observed under light microscope (Leica-DM2000LED) and documented.

### DNA extraction and DNA fragmentation assay by agarose gel electrophoresis

DNA is isolated from all the earthworm groups, including control worms, UV-C 1, 2, 3, and 5 min-treated groups. In brief, the small pieces of tissues are taken and washed with ice-cold PBS buffer. Then it is taken in the vial and added with 50 μl of digestion buffer, homogenized and further incubated at 50 °C for 6–12 h. Following incubation, homogenized tissues are added with equal amount of a mixture of phenol, chloroform, and isopropyl alcohol in the ratio of (25:24:1) and kept in a rocker for 10 min. At that point, transfer the aqueous layer to a fresh tube and add half the volume of ammonium acetate and double the volume of 100% ethanol. Later, centrifuge this mixture at 10,000 rpm for 2 min. Subsequently, remove the supernatant and add 700 μl of 70% ethanol to the pellet and mix well. Repeat the step again and dry the pellet for a few minutes. Finally, add 50 μl of 1 × TE buffer to the dried pelleted DNA, mix well, and store it at 4 °C. The isolated DNA is in 1% agarose gel electrophoresis. DNA bands are analyzed and compared with the control group.

### Comet assay

#### Cell isolation

The earthworm tissue is washed with ice cold water and cut into very small tissue fragments. Then the cell is dissociated from the tissue fragment in ice cold PBS containing 50 mM EDTA and 0.1% trypsin by using fine needles. Further, transfer the cell suspension into a centrifuge tube and allow to stand it for 5 min at room temperature to settle down the debris. Subsequently, the supernatant is collected by centrifugation at 1000 rpm for 4 min, the supernatant is discarded and the pellet is added with ice cold PBS. Repeat the step once again and collect the cell and mix it well with ice cold PBS and keep at 4 °C in dark condition.

#### Slide preparation

The Comet slide base layer is coated with the 0.75% low melting agarose on frosted glass slide at a temperature of 40 °C. Then it is kept at 4 °C for 15 min. Later, the earthworm cell suspension is mixed with low melting agarose in 1:10 ratio at 37 °C. Subsequently, 100 μl of this suspension is taken and coated on the base layer of the prepared slide and allowed to dry at 4 °C for 15 min in dark condition.

Further, it is treated with lysis buffer (contains triton × 100) for 1 h followed by incubation in alkaline solution for 30 min in ice cold condition. Then it is run in the electrophoresis at 35 V for 15 min. Later slides are transferred into pre-chilled water and immersed for 2 min twice. Later, transfer it to pre-chilled 70% ethanol for 5 min and allow it to dry in a horizontal position. Finally, the slides are incubated with Bisbenzimide Hoechst 3342 fluorescent stain for 15 min followed by documentation using Evos inverted fluorescence microscope.

### SDS PAGE and immunoblotting

The earthworm, *P. excavatus* tissues are taken for the protein sample preparation. Briefly, tissues are homogenized with 2 × protein sample buffer and the homogenate are boiled for 5 min. Subsequently, 10 μg of each protein samples are resolved in 12% SDS-PAGE. The resolved proteins in the SDS-PAGE gel are transferred to the PVDF membrane. The membrane is then incubated with a primary antibody, H2AX (Rabbit polyclonal, Abcam-ab20669), and β-Actin (Mouse monoclonal, Abcam-ab8227) at the dilution of 1:5000 at 4 °C for 6 h. Following incubation, the membrane is washed with 1 × PBST buffer thrice and incubated with secondary antibody, anti-rabbit IgG, HRP (produced in goat, Sigma-A0545), and Anti-mouse IgG, HRP (produced in goat Abcam-ab6789) at a dilution of 1:10,000 for 2 h at room temperature. Following the washing step, the band signals in the membrane are developed using an X-ray film. The ECL is a chemiluminescent substrate used for the detection of horseradish peroxidase (HRP). After incubating the membrane in ECL substrate solution for 1 min, the membrane is removed and excess liquid is drained out. Then the membrane is placed inside the transparent plastic sheet and positioned inside the cassette followed by situating an X-ray film over it to close the cassette to capture the signals. After 5 min of exposure, the X-ray film is immersed few times in the developer solution and the signals in the X-ray film are developed followed by immersion in water. Later, immerse it in fixture solution followed by water. Then, the X-ray film is allowed to dry for documentation.

### Statistical analysis

Survival graph (Kaplan–Meier) for the UV-C-treated earthworms is plotted using the statistical software—GraphPad Prism, Version 5.01. Briefly, UV-C treatment of the earthworms, death, and live worms are counted and plotted in the (Kaplan–Meier) survival graph. All other experiments are repeated for at least three times to obtain the statistical significance. The data obtained were analysed using the statistical software SPSS (version 22.0; IBM Corp., USA) and expressed as mean ± SD. The results were considered as statistical significance when the *p*-value < 0.05.

## Supplementary information

Supplementary Video 1.

Supplementary Information 1.

Supplementary Information 2.

## Data Availability

All data generated or analyzed during this study are included in this published article.

## References

[1] Hoeijmakers JHJ (2009). DNA damage, aging, and cancer. N. Engl. J. Med..

[2] Roy S, Choudhury SR, Singh SK, Das KP (2011). AtPolλ, a homolog of mammalian DNA polymerase λ in Arabidopsis thaliana, is involved in the repair of UV-B induced DNA damage through the dark repair pathway. Plant Cell Physiol..

[3] MacRae SL (2015). DNA repair in species with extreme lifespan differences. Aging (Albany, NY).

[4] Gartner A, Milstein S, Ahmed S, Hodgkin J, Hengartner MO (2000). A conserved checkpoint pathway mediates DNA damage-induced apoptosis and cell cycle arrest in *C. elegans*. Mol. Cell.

[5] O’Driscoll M, Jeggo PA (2006). The role of double-strand break repair—insights from human genetics. Nat. Rev. Genet..

[6] Stucki M, Jackson SP (2006). γH2AX and MDC1: anchoring the DNA-damage-response machinery to broken chromosomes. DNA Repair (Amst).

[7] Thiriet C, Hayes JJ (2005). Chromatin in need of a fix: phosphorylation of H2AX connects chromatin to DNA repair. Mol. Cell.

[8] Nussenzweig A, Paull T (2006). DNA repair: tails of histones lost. Nature.

[9] Ünal E (2004). DNA damage response pathway uses histone modification to assemble a double-strand break-specific cohesin domain. Mol. Cell.

[10] Sinha RP, Häder D-P (2002). UV-induced DNA damage and repair: a review. Photochem. Photobiol. Sci..

[11] Guerrero-Beltr-n JA, Barbosa-C· novas GV (2004). Advantages and limitations on processing foods by UV light. Food Sci. Technol. Int..

[12] Steeger H-U, Freitag JF, Michl S, Wiemer M, Paul RJ (2001). Effects of UV-B radiation on embryonic, larval and juvenile stages of North Sea plaice (*Pleuronectes platessa*) under simulated ozone-hole conditions. Helgol. Mar. Res..

[13] Misra RB, Babu GS, Ray RS, Hans RK (2002). Tubifex: a sensitive model for UV-B-induced phototoxicity. Ecotoxicol. Environ. Saf..

[14] Bais AF (2018). Environmental effects of ozone depletion, UV radiation and interactions with climate change: UNEP Environmental Effects Assessment Panel, update 2017. Photochem. Photobiol. Sci..

[15] Dixon AJ, Dixon BF (2004). Ultraviolet radiation from welding and possible risk of skin and ocular malignancy. Med. J. Aust..

[16] Welch D (2018). Far-UVC light: A new tool to control the spread of airborne-mediated microbial diseases. Sci. Rep..

[17] Wu J (2017). The influence of postharvest UV-C treatment on anthocyanin biosynthesis in fresh-cut red cabbage. Sci. Rep..

[18] Bintsis T, Litopoulou-Tzanetaki E, Robinson RK (2000). Existing and potential applications of ultraviolet light in the food industry—a critical review. J. Sci. Food Agric..

[19] Manuela B, Welch D, Igor S, Brenner DJ (2020). Far-UVC light (222 nm) efficiently and safely inactivates airborne human coronaviruses. Sci. Rep..

[20] Pourzand C, Tyrrell RM (1999). Apoptosis, the role of oxidative stress and the example of solar UV radiation. Photochem. Photobiol..

[21] Davies RJH (1995). Ultraviolet radiation damage in DNA. Biochem. Soc. Trans..

[22] Fernandez-Capetillo O, Lee A, Nussenzweig M, Nussenzweig A (2004). H2AX: the histone guardian of the genome. DNA Repair (Amst)..

[23] Celeste A (2002). Genomic instability in mice lacking histone H2AX. Science.

[24] Pilch DR (2003). Characteristics of γ-H2AX foci at DNA double-strand breaks sites. Biochem. Cell Biol..

[25] Hanasoge S, Ljungman M (2007). H2AX phosphorylation after UV irradiation is triggered by DNA repair intermediates and is mediated by the ATR kinase. Carcinogenesis.

[26] Diffey BL (1991). Solar ultraviolet radiation effects on biological systems. Phys. Med. Biol..

[27] Modenese A, Korpinen L, Gobba F (2018). Solar radiation exposure and outdoor work: an underestimated occupational risk. Int. J. Environ. Res. Public Health..

[28] Edwards CA, Bohlen PJ (1996). Biology and ecology of earthworms.

[29] Johnson Retnaraj Samuel SC (2011). Autofluorescence in BrdU-positive cells and augmentation of regeneration kinetics by riboflavin. Stem Cells Dev..

[30] Yesudhason BV (2018). Exploiting the unique phenotypes of the earthworm *Eudrilus eugeniae* to evaluate the toxicity of chemical substances. Environ. Monit. Assess..

[31] Engelmann P, Pál J, Berki T, Cooper EL, Németh P (2002). Earthworm leukocytes react with different mammalian antigen-specific monoclonal antibodies. Zoology.

[32] Płytycz B, Homa J, Kozioł B, Rózanowska M, Morgan AJ (2006). Riboflavin content in autofluorescent earthworm coelomocytes is species-specific. Folia Histochem. Cytobiol..

[33] Clydesdale GJ, Dandie GW, Muller HK (2001). Ultraviolet light induced injury: immunological and inflammatory effects. Immunol. Cell Biol..

[34] Banaszak AT, Trench RK (1995). Effects of ultraviolet (UV) radiation on marine microalgal-invertebrate symbioses. II. The synthesis of mycosporine-like amino acids in response to exposure to UV in *Anthopleura **elegantissima* and *Cassiopeia **xamachana*. J. Exp. Mar. Biol. Ecol..

[35] Carefoot TH, Harris M, Taylor BE, Donovan D, Karentz D (1998). Mycosporine-like amino acids: possible UV protection in eggs of the sea hare *Aplysia **dactylomela*. Mar. Biol..

[36] Inal ME, Kahraman A (2000). The protective effect of flavonol quercetin against ultraviolet a induced oxidative stress in rats. Toxicology.

[37] Chuang S-C, Lai W-S, Chen J-H (2006). Influence of ultraviolet radiation on selected physiological responses of earthworms. J. Exp. Biol..

[38] Guggiana-Nilo DA, Engert F (2016). Properties of the visible light phototaxis and UV avoidance behaviors in the larval zebrafish. Front. Behav. Neurosci..

[39] Hess WN (1925). Nervous system of the earthworm, *Lumbricus **terrestris* L. J. Morphol..

[40] Holm-Hansen, O., Lubin, D. & Helbling, E. W. Ultraviolet radiation and its effects on organisms in aquatic environments. In* Environmental UV photobiology *379–425 (Springer, 1993).

[41] Soni AK, Joshi PC (1997). High sensitivity of Tubifex for ultraviolet-B. Biochem. Biophys. Res. Commun..

[42] van de Mortel TF, Buttemer WA (1998). Avoidance of ultraviolet-B radiation in frogs and tadpoles of the species *Litoria aurea*, *L. dentata* and *L, peronii*. Proc. Linn. Soc. New South Wales.

[43] Banerjee S, Leptin M (2014). Systemic response to ultraviolet radiation involves induction of leukocytic IL-1β and inflammation in zebrafish. J. Immunol..

[44] Rana S, Byrne SN, MacDonald LJ, Chan CY-Y, Halliday GM (2008). Ultraviolet B suppresses immunity by inhibiting effector and memory T cells. Am. J. Pathol..

[45] Sasaki N (2017). UVB exposure prevents atherosclerosis by regulating immunoinflammatory responses. Arterioscler. Thromb. Vasc. Biol..

[46] Lesser MP, Farrell JH, Walker CW (2001). Oxidative stress, DNA damage and p53 expression in the larvae of Atlantic cod (*Gadus**morhua*) exposed to ultraviolet (290–400 nm) radiation. J. Exp. Biol..

[47] Singh MK, Sharma JG, Chakrabarti R (2015). Simulation study of natural UV-B radiation on *Catla**catla* and its impact on physiology, oxidative stress, Hsp 70 and DNA fragmentation. J. Photochem. Photobiol. B Biol..

[48] Ramírez-Duarte WF, Kurobe T, Teh SJ (2017). Effects of low levels of ultraviolet radiation on antioxidant mechanisms of Japanese Medaka (*Oryzias**latipes*). Chemosphere.

[49] Albro PW, Bilski P, Corbett JT, Schroeder JL, Chignell CF (1997). Photochemical reactions and phototoxicity of sterols: novel self-perpetuating mechanism for lipid photooxidation. Photochem. Photobiol..

[50] Morita A, Krutmann J (2000). Ultraviolet A radiation-induced apoptosis. Methods Enzymol..

[51] Tang D, Kang R, Berghe TV, Vandenabeele P, Kroemer G (2019). The molecular machinery of regulated cell death. Cell Res..

[52] Godar DE, Lucas AD (1995). Spectral dependence of UV-induced immediate and delayed apoptosis: the role of membrane and DNA damage. Photochem. Photobiol..

[53] Maginnis TL (2006). The costs of autotomy and regeneration in animals: a review and framework for future research. Behav. Ecol..

[54] Cooper EL (2018). Advances in comparative immunology.

[55] Rinkevich B, Müller WE (2012). Invertebrate immunology.

[56] Wakasugi M (2014). Nucleotide excision repair-dependent DNA double-strand break formation and ATM signaling activation in mammalian quiescent cells. J. Biol. Chem..

[57] Azzouz D, Khan MA, Sweezey N, Palaniyar N (2018). Two-in-one: UV radiation simultaneously induces apoptosis and NETosis. Cell Death Discov..

[58] Lee CH, Wu SB, Hong CH, Yu HS, Wei YH (2013). Molecular mechanisms of UV-induced apoptosis and its effects on skin residential cells: the implication in UV-based phototherapy. Int. J. Mol. Sci..

[59] Araldi RP (2015). Using the comet and micronucleus assays for genotoxicity studies: a review. Biomed. Pharmacother..

[60] Nagata S, Nagase H, Kawane K, Mukae N, Fukuyama H (2003). Degradation of chromosomal DNA during apoptosis. Cell Death Differ..

[61] Zhang JH, Ming XU (2000). DNA fragmentation in apoptosis. Cell Res..

[62] Rogakou EP, Nieves-Neira W, Boon C, Pommier Y, Bonner WM (2000). Initiation of DNA fragmentation during apoptosis induces phosphorylation of H2AX histone at serine 139. J. Biol. Chem..

[63] Turinetto V, Giachino C (2015). Survey and summary multiple facets of histone variant H2AX: a DNA double-strand-break marker with several biological functions. Nucleic Acids Res..

[64] Friedberg EC, Walker GC, Siede W, Wood RD (2005). DNA repair and mutagenesis.

[65] Marti TM, Hefner E, Feeney L, Natale V, Cleaver JE (2006). H2AX phosphorylation within the G1 phase after UV irradiation depends on nucleotide excision repair and not DNA double-strand breaks. Proc. Natl. Acad. Sci. U.S.A..

[66] O'Driscoll M, Ruiz-Perez VL, Woods CG, Jeggo PA, Goodship JA (2003). A splicing mutation affecting expression of ataxia–telangiectasia and Rad3-related protein (ATR) results in Seckel syndrome. Nat. Genet..

[67] Caldecott KW (2008). Single-strand break repair and genetic disease. Nat. Rev. Genet..

[68] Kastan MB, Lim DS (2000). The many substrates and functions of ATM. Nat. Rev. Mol. Cell Biol..

[69] Ward IM, Chen J (2001). Histone H2AX is phosphorylated in an ATR-dependent manner in response to replicational stress. J. Biol. Chem..

[70] Wang H, Wang M, Wang H, Böcker W, Iliakis G (2005). Complex H2AX phosphorylation patterns by multiple kinases including ATM and DNA-PK in human cells exposed to ionizing radiation and treated with kinase inhibitors. J. Cell. Physiol..

[71] Oh K-S, Bustin M, Mazur SJ, Appella E, Kraemer KH (2011). UV-induced histone H2AX phosphorylation and DNA damage related proteins accumulate and persist in nucleotide excision repair-deficient XP-B cells. DNA Repair (Amst)..

[72] Wakasugi M (2014). Nucleotide excision repair-dependent DNA double-strand break formation and ATM signaling activation in mammalian quiescent cells. J. Biol. Chem..

[73] Li L, Yan Y, Xu H, Qu T, Wang B (2011). Selection of reference genes for gene expression studies in ultraviolet B-irradiated human skin fibroblasts using quantitative real-time PCR. BMC Mol. Biol..

[74] Miller AC (2002). Proto-oncogene expression: a predictive assay for radiation biodosimetry applications. Radiat. Prot. Dosim..

[75] Amundson SA (2004). Human in vivo radiation-induced biomarkers: gene expression changes in radiotherapy patients. Cancer Res..

[76] Sharungbam GD (2012). Identification of stable endogenous control genes for transcriptional profiling of photon, proton and carbon-ion irradiated cells. Radiat. Oncol..

[77] Novo M, Muñiz-González AB, Trigo D, Casquero S, Guitarte JLM (2019). Applying sunscreens on earthworms: Molecular response of *Eisenia **fetida* after direct contact with an organic UV filter. Sci. Total Environ..

