# Publisher Correction: Exploring the effect of UV-C radiation on earthworm and understanding its genomic integrity in the context of H2AX expression

**DOI:** 10.1038/s41598-021-85417-w

**Published:** 2021-03-11

**Authors:** Karthikeyan Subbiahanadar Chelladurai, Jackson Durairaj Selvan Christyraj, Ananthaselvam Azhagesan, Vennila Devi Paulraj, Muralidharan Jothimani, Beryl Vedha Yesudhason, Niranjan Chellathurai Vasantha, Mijithra Ganesan, Kamarajan Rajagopalan, Saravanakumar Venkatachalam, Johnson Benedict, Jemima Kamalapriya John Samuel, Johnson Retnaraj Samuel Selvan Christyraj

**Affiliations:** 1 Regeneration and Stem Cell Biology Lab, Centre for Molecular and Nanomedical Sciences, International Research Centre, Sathyabama Institute of Science and Technology, Chennai, 600119 Tamilnadu India; 2 Present Address: Centre for Nanobiotechnology, Vellore Institute of Technology, Vellore, 632014 Tamilnadu India; 3 Present Address: Department of Bioinformatics, Science Campus, Alagappa University, Karaikudi, 630004 Tamilnadu India; 4 Department of Biotechnology, Anna University of Technology, Tiruchirappalli, 620024 Tamilnadu India

Correction to: *Scientific Reports* 10.1038/s41598-020-77719-2, published online 03 December 2020

In the original version of this Article, Muralidharan Jothimani was incorrectly affiliated with ‘Present Address: Centre for Nanobiotechnology, Vellore Institute of Technology, Vellore 632014, Tamilnadu, India’. The correct affiliations are listed below.

Regeneration and Stem Cell Biology Lab, Centre for Molecular and Nanomedical Sciences, International Research Centre, Sathyabama Institute of Science and Technology, Chennai 600119, Tamilnadu, India

Present address: Department of Bioinformatics, Science Campus, Alagappa University, Karaikudi 630004, Tamilnadu, India.

This error has now been corrected in the PDF and HTML versions of the Article.

