# Supplementary material for: Exploring the effect of UV-C radiation on earthworm and understanding its genomic integrity in the context of H2AX expression
Source: Sci Rep. 2020 Dec 3;10:21005. doi: 10.1038/s41598-020-77719-2 (PMC7713072; doi:10.1038/s41598-020-77719-2)
Supplement: Supplementary file 2 — Supplementary Information 1. [file 41598_2020_77719_MOESM2_ESM.docx]

**Exploring the effect of UV-C radiation on earthworm and understanding its genomic integrity in the context of H2AX expression**

Subbiahanadar Chelladurai Karthikeyan, Selvan Christyraj Jackson Durairaj*, Azhagesan Ananthaselvam, Vennila Devi Paulraj, Muralidharan Jothimani, Beryl Vedha Yesudhason, Niranjan Chellathurai Vasantha, Mijithra Ganesan, Kamarajan Rajagopalan, Saravanakumar Venkatachalam, Johnson Benedict, Jemima Kamalapriya John Samuel, Johnson Retnaraj Samuel Selvan Christyraj*

**Supplementary data: 1**

**UV dose calculation**

Exposed area is 120 x 60 x 55 cm, wavelength of that UV-C light is 253.7nm. Duration of UV exposure for different time period 1, 2, 3 and 5 minutes.

Therefore, the UV doses calculated as follows

**E=hc/λ**

h= Planck constant (6.626× 10-34m^2^ kg/s)

c=Speed of light in vacuum (3×108m/s)

λ (lambda)= Wavelength of the radiation= 253.7nm (which is 2.53×10^-7^m)

So, E= 6.626×10^-34^× (3×10^8^)/2.53×10^-7^

= 7.856×10-19

Units = [(m^2^ kg/ s) (m/s)] (m) = (m^2^ kg/s × m/s) × (1/m) = m^2^ kg/s^2^ =J

Therefore,

E = 7.856 ×10-19 J

Irradiation duration= 1, 2, 3 and 5 minutes.

For, 1minutes= 60seconds

E= 7.856 ×10-19× 60

= 4.714 × 10^-17^ J

Distance between UV light and the surface = 55cm

Consideration of squared law

55cm = 0.55 (0.55)2 = 0.3025m^2^

Divide the above equation by 1/0.3025 = 3.305m^2^

E= 4.714×10^-17^ J / 3.305 m^2^

= 1.426×10^-17^ J/ m^2^

Therefore, for UV dose for 1min = **1.426×10^-17^ J/ m^2^**

Similarly,

UV dose for 2min =**2.85 x 10^-17^ J/m^2^**

UV dose for 3min= **4.27 x 10^-17^ J/m^2^**

UV dose for 5min= **7.13 x 10^-17^ J/m^2^**
