# Supplementary figures and images for: Exploring the effect of UV-C radiation on earthworm and understanding its genomic integrity in the context of H2AX expression
Source: Sci Rep. 2020 Dec 3;10:21005. doi: 10.1038/s41598-020-77719-2 (PMC7713072; doi:10.1038/s41598-020-77719-2)

**Original raw data**

DNA fragmentation assay


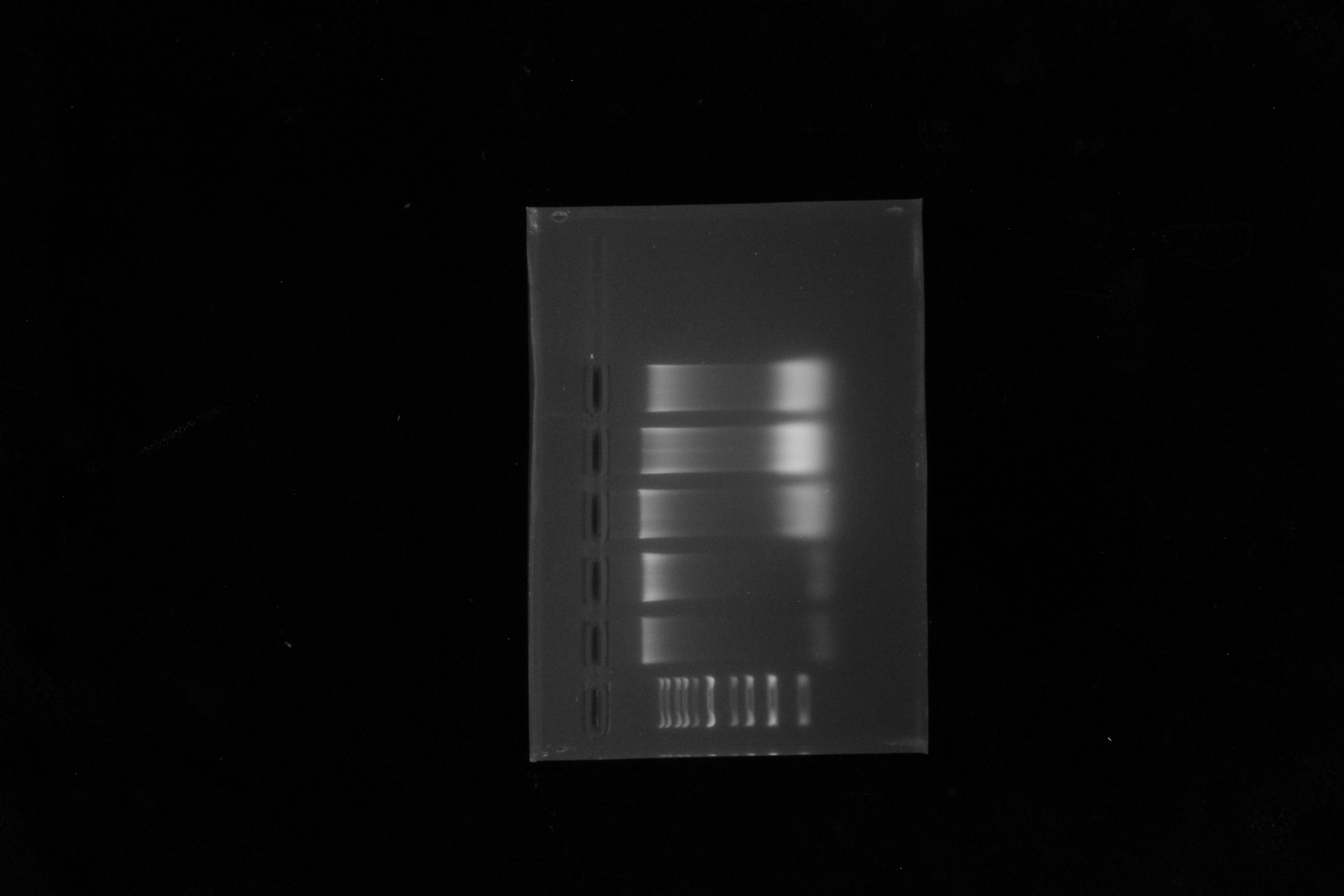


**Immunoblot**

1.
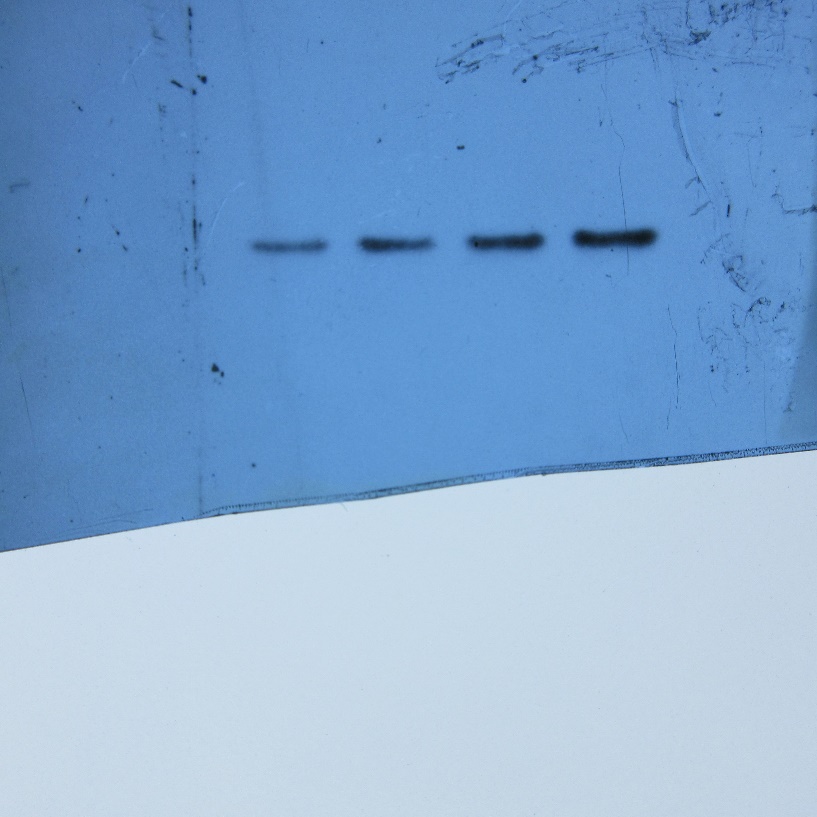
**H2AX**
2. **Beta-actin**


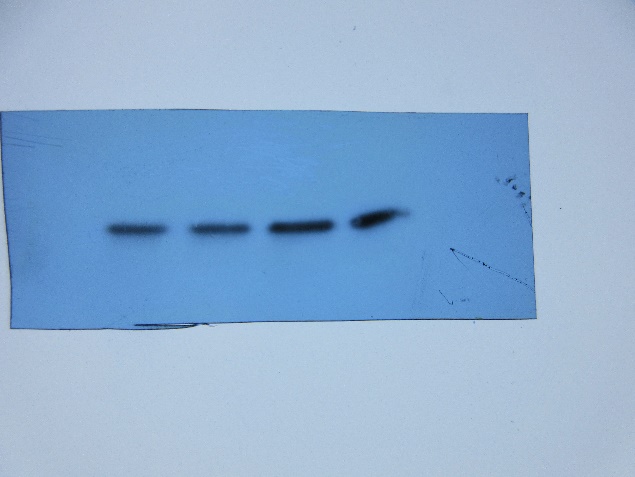

Supplement: Supplementary file 3 — Supplementary Information 2. [file 41598_2020_77719_MOESM3_ESM.docx]
